# Cost-of-illness studies in rare diseases: a scoping review

**DOI:** 10.1186/s13023-021-01815-3

**Published:** 2021-04-13

**Authors:** Lidia García-Pérez, Renata Linertová, Cristina Valcárcel-Nazco, Manuel Posada, Inigo Gorostiza, Pedro Serrano-Aguilar

**Affiliations:** 1 Servicio de Evaluación del Servicio Canario de la Salud (SESCS), Camino Candelaria Nº 44, 1ª planta, 38109 Canary Islands, El Rosario, Santa Cruz de Tenerife, Spain; 2 Fundación Canaria Instituto de Investigación Sanitaria de Canarias (FIISC), Camino Candelaria Nº 44, 1ª planta, 38109 Canary Islands, El Rosario, Santa Cruz de Tenerife, Spain; 3 Red de Investigación en Servicios de Salud en Enfermedades Crónicas (REDISSEC), Madrid, Spain; 4 Instituto Universitario de Desarrollo Regional (IUDR), Universidad de La Laguna, Campus de Guajara, Camino de la Hornera, s/n, 38071 La Laguna, Santa Cruz de Tenerife, Spain; 5 Centro de Investigaciones Biomédicas de Canarias (CIBICAN), Universidad de La Laguna, La Laguna, Spain; 6 Institute of Rare Diseases Research, Institute of Health Carlos III, Monforte de Lemos, 5, 28029 Madrid, Spain; 7 CIBER of Rare Diseases (CIBERER), Madrid, Spain; 8 Osakidetza Basque Health Service, Basurto University Hospital, Avenida de Montevideo Nº 18, 48013 Bilbao, Spain

**Keywords:** Cost-of-illness, Economic burden, Rare diseases, Scoping review

## Abstract

**Objective:**

The aim of this scoping review was to overview the cost-of-illness studies conducted in rare diseases.

**Methods:**

We searched papers published in English in PubMed from January 2007 to December 2018. We selected cost-of-illness studies on rare diseases defined as those with prevalence lower than 5 per 10,000 cases. Studies were selected by one researcher and verified by a second researcher. Methodological characteristics were extracted to develop a narrative synthesis.

**Results:**

We included 63 cost-of-illness studies on 42 rare diseases conducted in 25 countries, and 9 systematic reviews. Most studies (94%) adopted a prevalence-based estimation, where the predominant design was cross-sectional with a bottom-up approach. Only four studies adopted an incidence-based estimation. Most studies used questionnaires to patients or caregivers to collect resource utilisation data (67%) although an important number of studies used databases or registries as a source of data (48%). Costs of lost productivity, non-medical costs and informal care costs were included in 68%, 60% and 43% of studies, respectively.

**Conclusion:**

This review found a paucity of cost-of-illness studies in rare diseases. However, the analysis shows that the cost-of-illness studies of rare diseases are feasible, although the main issue is the lack of primary and/or aggregated data that often prevents a reliable estimation of the economic burden.

**Supplementary Information:**

The online version contains supplementary material available at 10.1186/s13023-021-01815-3.

## Introduction

Rare diseases (RD) are those that affect a small part of the population. A disease can be rare in one region and more prevalent in another, so there are different definitions and classifications. In Europe, the European Union Regulation on Orphan Medicinal Products (1999) defines a RD as a disease that affects not more than 1 person per 2000 in the population [1]. Although the number of patients with a specific RD is low, the sum of people affected by any RD is large. More than 6 thousand RDs have been reported and it is estimated that they could affect about 29 million people in the European Union [2]. Similar to other health conditions, RDs impose a high social and economic burden for patients and their families, health care systems and society overall. Research in this field is key for progress in the knowledge of RDs in general [3] and for the sustainability of health care systems [4].

The economic burden of a disease is measured through cost-of-illness (COI) studies, and defined as the ‘maximum amount that could potentially be saved or gained if a disease were to be eradicated’ [5]. The estimation of this amount consists of three phases: identification, quantification and valuation in monetary terms of all resources associated with a disease. A COI study can be addressed by means of different designs and points of view. The perspective adopted to perform a COI study can be that of society, government, healthcare system, third party payer, employers or families. The type of perspective implies the inclusion of different types of costs which can be classified into three main categories: direct medical costs, direct non-medical costs and costs due to productivity losses (also called indirect costs).

COI studies can use either prevalence-based or incidence-based estimations and their design is usually a mathematical model or a cross-sectional study, either prospective or retrospective. Moreover, there are different approaches to quantify resources and calculate costs: a top-down approach, a bottom-up approach or a mixed approach. The selection of the characteristics of the COI study depends on the objective, disease and practical reasons, such as the available time and resources to conduct the study, as some designs are costlier than others.

COI studies enable comparison between countries and between diseases [6] so it is useful to learn about the relative burden of a RD in comparison to other RDs or non-rare diseases. Continuous or periodic monitoring of the COI would enable ascertaining the evolution of the burden for a country over time and, consequently, the effect of policies and new medical developments. In fact, COI studies can be a tool to prioritize diseases, their prevention and treatment, or to implement other non-healthcare policies, such as benefits for patients and families/caregivers. In this regard, COI studies are one of the methods that meets the third goal of the International Rare Diseases Research Consortium (www.irdirc.org), that is, methods to assess the impact of diagnoses and therapies on RDs and, consequently, they must be part of the research agenda in the field of RDs.

Despite the relevance of this type of studies and the value of their results, a lack of COI studies focused on RDs is commonly acknowledged [7]. With the objective of depicting the state-of-the-art and the methodological characteristics of the COI in RDs, a scoping review of published literature was conducted and its results discussed following a systematic approach to research and report [8].

## Methods

### Eligibility criteria

We selected COI studies of RDs where the diseases had a reported prevalence lower than 5 per 10,000 cases according to the Orphanet database (www.orpha.net) or the most recent Orphanet’s report [9], independently of the country where the research was conducted. To be consistent with the definition of COI, we included studies that at least estimated medical costs (even when the perspective was limited: only hospital perspective or only patient perspectives); consequently we excluded those studies that only estimated the cost of lost productivity. We also included systematic reviews of COI studies of RDs. We excluded case reports, conferences proceedings, and partial or complete economic evaluations of technologies; we also excluded studies on rare cancers, infectious diseases, comorbidities and/or adverse events.

### Information sources

An initial search was conducted in April 2017; the definitive search was conducted in PubMed in January 2020 and included references published in English from January 2007 to December 2018. Editorials, letters and references without abstracts were excluded. Given the variety of RDs and the non-sensitiveness of the MeSH for RDs for our purpose, we conducted a search strategy where only cost-related terms were used, that is, without disease-related terms. To increase the specificity of our results, we searched for studies already indexed with the ‘cost-of-illness’ MeSH (see Table 1).

**Table 1:** Search strategy in PubMed

((((cost[Title] OR costs[Title] OR ((socioeconomic[Title] OR economic[Title]) AND burden[Title]) OR "cost of illness"[Title]))) AND ("cost of illness"[MeSH])) NOT (("editorial"[Publication Type] OR "letter"[Publication Type])) Filters: Abstract; Publication date from 2007/01/01 to 2018/12/31; Humans; English	

### Selection of sources of evidence, data charting process and synthesis of results

One researcher (economist) screened the titles and abstracts. A second researcher (economist or physician) verified the selection, and disagreements were resolved by consensus. Characteristics of the selected studies were extracted, that is, country, disease or group of diseases, sample size, design, prevalence or incidence-based estimation, bottom-up or top-down approach, sources of patients and data, time horizon and discount, perspective and costs included, method for the estimation of productivity costs. Given the purpose of this review, we did not extract cost estimates nor did we assess the quality of the studies. We present a narrative synthesis.

## Results

### Selection of sources of evidence

**Fig. 1:**
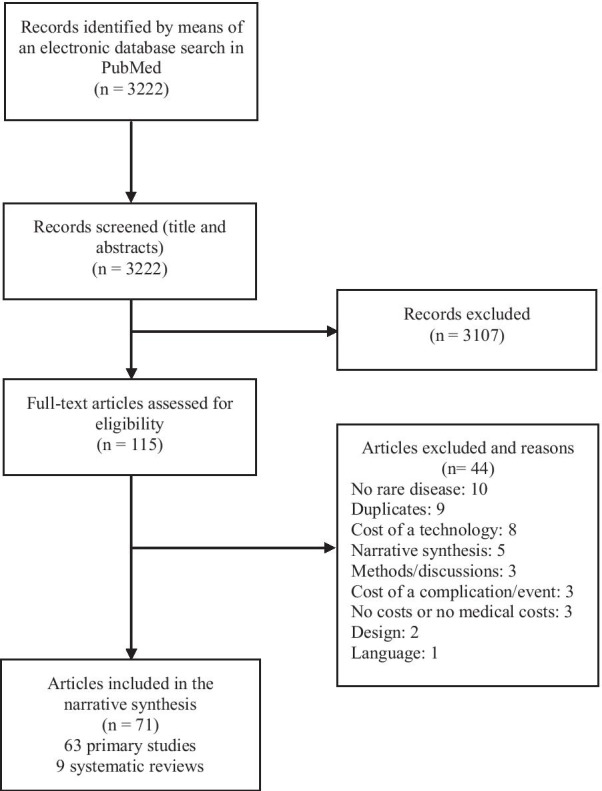
Flow diagram of study selection

The search identified 3222 references. After conducting an initial screening of titles and abstracts, 3107 were excluded. Given the non-specific search strategy, most excluded references during this phase focused on non-rare disease. Consequently, 115 full-text articles were retrieved and assessed for eligibility. Forty four out of 115 references were excluded due to several reasons (see Fig. 1). The remaining 71 articles were selected for inclusion in this scoping review, 63 COI studies [10–72] and 9 systematic reviews [7, 30, 73–79] (one of the articles included a COI study and a systematic review [30]).

### Characteristics of sources of evidence and synthesis of results

Table 2 shows the 63 included studies covering 42 RDs. Ten diseases were studied in more than one study (amyotrophic lateral sclerosis (ALS), haemophilia, Duchenne muscular dystrophy, cystic fibrosis, chronic inflammatory demyelinating polyradiculoneuropathy, idiopathic pulmonary fibrosis, juvenile idiopathic arthritis (JIA), Prader-Willi syndrome, systemic sclerosis, tuberous sclerosis complex); another 32 diseases were subject of only one study.

**Table 2:** Included cost-of-illness studies per disease

Disease or group of diseases	Prevalence	Number of studies	References
Amyotrophic lateral sclerosis (ALS)	1–9/100,000	8	[11, 12, 19, 24, 34, 41, 48, 62]
Haemophilia	1–9/100,000	7	[21, 28, 44, 49, 51, 66, 69]
Duchenne muscular dystrophy	1–9/100,000	5	[34, 54, 55, 60, 61]
Cystic fibrosis	1–9/100,000	4	[30, 46, 63, 71]
Chronic inflammatory demyelinating polyradiculoneuropathy	1–9/100,000	3	[13, 42, 43]
Idiopathic pulmonary fibrosis	1–5/10,000	2	[68, 72]
Juvenile idiopathic arthritis (JIA)	1–9/100,000	2	[31, 45]
Prader-Willi syndrome	1–9/100,000	2	[37, 56]
Systemic sclerosis	1–5/10,000	2	[22, 38]
Tuberous sclerosis complex	1–5/10,000	2	[29, 58]
Achalasia (Idiopathic achalasia)	1–9/100,000	1	[47]
Acromegaly	1–9/100,000	1	[35]
Autosomal dominant polycystic kidney disease	1–5/10,000	1	[16]
Becker muscular dystrophy	1–9/100,000	1	[54]
Behçet’s syndrome	1–9/100,000	1	[59]
Common variable immunodeficiency	1–9/100,000	1	[53]
Congenital hyperinsulinism	1–9/100,000	1	[15]
Cushing disease	1–9/100,000	1	[33]
Dermatomyositis	1–9/100,000	1	[32]
Dravet syndrome	1–9/100,000	1	[64]
Epidermolysis bullosa	1–9/100,000	1	[10]
Fragile x syndrome	1–5/10,000	1	[70]
Frontotemporal degeneration	1–5/10,000	1	[18]
Guillain-Barré syndrome	1–9/100,000	1	[17]
Hereditary angioedema	1–9/100,000	1	[65]
Histiocytosis	1–9/100,000	1	[27]
Lysosomal acid lipase deficiency, Cholesteryl ester storage disease type	1–9/100,000	1	[20]
Mucopolysaccharidosis	Depends on the type*	1	[50]
Multifocal motor neuropathy	1–9/100,000	1	[42]
Multiple system atrophy	1–9/100,000	1	[67]
Myotonic dystrophy	1–9/100,000	1	[34]
Narcolepsy-cataplexy syndrome	1–5/10,000	1	[26]
Niemann-Pick disease type C	1–9/100,000	1	[25]
Paraproteinaemic demyelinating neuropathy	1–9/100,000	1	[42]
Pemphigus	Depends on the type **	1	[23]
Phenylketonuria (PKU)	1–5/10,000	1	[14]
Primary childhood glaucoma and secondary childhood glaucoma	1–9/100,000	1	[36]
Progressive supranuclear palsy	1–9/100,000	1	[67]
Pulmonary arterial hypertension	1–9/100,000	1	[57]
Sarcoidosis	1–5/10,000	1	[52]
Spinal muscular atrophy	1–9/100,000	1	[39]
Spinocerebellar ataxias	1–9/100,000	1	[40]

The sum is higher than 63 because 4 studies included more than one disease that fulfilled our inclusion criteria

^*^Type 1, Type 3, Type 6: 1–9/100,000; Type 2, Type 4: 1–5/10,000; Type 7: < 1 per million

^**^Superficial pemphigus: 1–9/100,000; pemphigus vulgaris: 1–5/10,000

Table 3 shows the characteristics of the COI studies (see Additional file 1 for more detailed characteristics). The studies retrieved were conducted in 25 countries: USA (19) and Canada (4) in America; in Europe: Germany (14), Spain (14), The United Kingdom (14), Italy (12), Sweden (12), France (11), Hungary (8), Bulgaria (7), The Netherlands (2), and another 11countries with only one paper (Belgium, Czech Republic, Denmark, Finland, Greece, Ireland, Norway, Poland); two studies were conducted in Australia and one in other 5 Asian countries: India, Iran, Korea, Taiwan and Turkey. The high number of studies in some European countries is due to the BURQOL-RD Study [80], which tackled the estimated burden of 10 RDs (cystic fibrosis, Duchenne muscular dystrophy, epidermolysis bullosa, fragile X syndrome, haemophilia, histiocytosis, JIA, mucopolysaccharidosis, Prader-Willi syndrome, and scleroderma) in Europe in 8 countries (Bulgaria, France, Germany, Hungary, Italy, Spain, Sweden, UK). Sample sizes ranged from as few as nine patients with multifocal motor neuropathy [42], 18 patients with Niemann-Pick disease type C [25] or 19 patients with lysosomal acid lipase deficiency [20], to larger samples, as 7855 patients with idiopathic pulmonary fibrosis [72], 7119 patients with sarcoidosis [52], or 6406 patients with pemphigus [23]. The studies with largest samples were conducted in the USA. Most diseases (78%) had a prevalence of 1–9 per 100,000 subjects.

**Table 3:** Characteristics of the cost-of-illness studies included in the review

Characteristic	Number of studies (%)
*Prevalence or incidence-based estimation*	
Prevalence	59 (94%)
Incidence	4 (6%)
*Design*	
Cross-sectional	40 (63%)
Cohort	10 (16%)
Cohort compared with a control cohort	7 (11%)
Mathematical model	5 (8%)
Cost study nested in a clinical trial	1 (2%)
*Prospective or retrospective*	
Retrospective	55 (87%)
Prospective	4 (6%)
*Source of patients*	
Registries or databases	32 (51%)
Hospital or other centres	24 (38%)
Patients’ organisations	18 (29%)
*Source of data*	
Questionnaires	42 (67%)
Registries or databases	30 (48%)
Other	4 (6%)
*Bottom-up or top-down approaches*	
Bottom-up	59 (94%)
Top-down	0 (0%)
Mixed	4 (6%)
*Time horizon*	
A year	52 (83%)
Lifetime	4 (6%)
Other	11 (17%)
*Perspective*	
Societal	40 (63%)
Third payer/health system/government	17 (27%)
Patients and families	7 (11%)
Hospital	3 (5%)
*Costs included in the analysis*	
Medical costs	63 (100%)
Non-medical costs	38 (60%)
Lost productivity costs	43 (68%)
Informal care costs	27 (43%)
*Method for the estimation of lost productivity costs*	
Human capital approach	34 (54%)
Friction cost approach	2 (3%)
Other	8 (13%)

Most studies (N = 59; 94%) adopted a prevalence-based estimation, where the predominant design was a retrospective cross-sectional design (40 studies). Nine studies followed a cohort design where 4 reported costs for several periods [20, 32, 36, 57] and 5 collected data along a period of time but only reporting the average cost per year [24, 46, 66, 68, 69]. In another 7 cohort studies patients with the disease where compared with a control cohort of subjects without the disease [13, 23, 29, 52, 56, 61, 72]. A cost study nested in a clinical trial [47] and two mathematical models [15, 53] were also included. All prevalence-based studies, except the mathematical models, adopted a bottom-up approach.

Only four studies (6%) adopted an incidence-based estimation approach with a patient’s lifetime horizon; all four discounted future costs to estimate the current value of costs. One of them had a longitudinal design reporting monthly costs in patients with ALS [12]. The other three studies developed mathematical models to estimate the lifetime cost of cystic fibrosis [63], haemophilia [21] and Guillain-Barré syndrome [17]. The last two studies adopted a mixed approach using individual data from administrative databases and aggregated data from statistics and literature [17, 21]. Both were able to populate the cost to estimate the national cost for the whole country. No studies using a pure top-down approach were identified.

The strategies to identify and access the patients were varied; eleven studies used more than one recruitment procedure. The studies recruited patients through patients’ organisations (29%), in hospitals and other centres (38%), or through access to databases or patient registries (51%). Some studies specifically recruited patients in reference centres or special units related to the disease [11, 46, 51, 69] or used specific disease registries [22, 54]. But an important number of studies used large databases such as health insurance claims databases [33], MEDICARE [72], or primary health care databases such as the Clinical Practice Research Datalink in the United Kingdom [29]. Other studies used social media associated to patients organizations to reach the patients [37, 50, 58].

Most studies collected resource utilisation data by means of questionnaires to patients or caregivers (67%), and/or extracting data from medical charts or other type of databases, such as public or private insurance claim data, registries or databases (48%). Questionnaires were self-reported by means of post, e-mail or online, or administered by telephone or face to face in a centre. As examples, questionnaires were designed ad hoc in some cases [25] or adapted from previous studies [42, 64]. They were usually administered once when patients were asked about resources utilisation during the last six months. Afterwards, these costs were doubled to estimate the annual costs incurred. Exceptionally, one study asked patients about the use of resources for the last three months, in two visits three months apart, so in the end the authors reported the cost of a six-month period [67]. Four studies collected data prospectively [47, 62, 66, 69]. One study administered a monthly questionnaire during the first year and a biannual questionnaire during the second year [69]. Another study gave patients a diary to prospectively collect the data and avoid the recall bias of retrospective questionnaires [62]; this way of collecting data is usually more expensive than asking retrospectively only once. Those studies where the data was only extracted from medical charts or databases did not include out of pocket costs, non-medical costs and indirect costs. Most studies estimated the annual cost per person (83%); the rest of studies reported costs using other time lengths such as one month, three months, half a year or several periods of years (17%).

A total of 16 studies (25%) attempted to estimate the costs for the overall population, generally assuming an approximate prevalence. Apart from the study by Lesen et al. that used a registry including all the population with acromegaly in Sweden [35] and the modelling studies [15, 17, 21, 53, 63], three other studies strived to estimate the national cost of certain diseases. Larkindale et al. calculated the total national costs in the USA of ALS, Duchenne muscular dystrophy and myotonic dystrophy, by multiplying the total per-patient cost (medical, nonmedical and indirect costs) by the prevalence of each disease reported by the Orphanet report and the best USA studies [34]. ALS had the largest estimated national cost, followed by Duchenne muscular dystrophy and myotonic dystrophy [34]. Similarly, O’Hara et al. extrapolated the costs obtained from their samples with haemophilia to country population by using national prevalence weights for 5 European countries [49]. Finally, Hsu et al. used the National Health Insurance Research Database that covers 99% of the population in Taiwan [24].

According to the costs included in the analysis, 63% of studies adopted the perspective of the society and 27% the health care system perspective as a third payer (MEDICARE, insurance companies, government); 11% of studies adopted a very restricted perspective including only costs incurred by patients, such as out-of-pocket or informal care. Three studies reported more than one perspective [25, 48, 57]. Imrie et al., for example, performed two analyses from both the societal and healthcare system perspectives, combining different sources of data. They selected patients from national databases which did not include health care data, but this lack of health data was offset by questionnaires designed ad hoc for patients [25].

All studies included medical costs (as this was an inclusion criterion), 60% included non-medical costs, 68% included the cost of lost productivity, and 43% included costs of informal care. Some studies classified informal care as a cost of lost productivity of parents or caregivers.

The method to estimate the cost of lost productivity was the “human capital approach” (HCA) and the “friction cost approach” (FCA) in 54% and 3% of studies, respectively. Other studies used other methods such as estimating difference in income before and after the disease or using the statistical value of a human life (13%). One study used two methods. Frenzen et al. used HCA for loss of productivity and the statistical value for premature deaths [17].

During the review we also identified eight systematic reviews on the costs of RDs: cystic fibrosis [30], Dravet syndrome [76], Duchenne muscular dystrophy [78], JIA [74], pulmonary arterial hypertension [75], systemic sclerosis [73], systemic vasculitis, i.e. Takayasu arteritis, Behçet’s syndrome, and Wegener’s granulomatosis [79], and the 10 diseases included in the BURQOL-RD project [7, 80].

## Discussion

The objective of this review was to show the state-of-the-art of the COI studies in the field of RDs. We found 63 papers that estimated the cost of 42 different RDs using several types of COI studies. Most studies were conducted in European countries, followed by the USA. Prevalence-based estimations are clearly preferred over incidence-based ones as it is usual with other type of diseases [5]. Nevertheless, we found four examples of incidence-based COI studies in our review, showing the feasibility of this approach for those diseases with sufficient information available for the relevant time horizon according to expected lifetime related to the disease.

Regarding the design, when we started this study in 2016, we only found one study where the cohort with the disease was compared with a group without the disease [72]. Collard et al. used claims data from MEDICARE to compare healthcare costs before and after the diagnosis of idiopathic pulmonary fibrosis [72]. After updating the review we found another 6 studies, published between 2016 and 2018, with this kind of design. This design has advantages to estimate real incremental costs as it enables estimation of the difference on costs between having and not having the diseases [81]. There are no reasons to think that this design is not possible for RDs, as it has been performed for more prevalent diseases [82]. However, it can be hypothesised that for some RDs, where the disease rules over the patient’s life, a huge part of health care resources could be directly related to the disease. Nonetheless, there are other methods to measure incremental costs, such as regression analysis or selection of those procedures or interventions directly related to the disease when collecting data from records or questionnaires [81].

Costs included in the analyses depend on the perspective or point of view of those using the information for making decisions, but the societal perspective is the most complete and the preferred among health economists [81, 83]. Although the perspective was not always explicitly stated, the societal perspective was also the most common among the COI studies on RDs; the third-party payer perspective, that is, a health insurance or the public health care perspective, was also used in a quarter of the studies. Per protocol, all studies included direct medical costs. Regarding indirect costs, HCA was the most commonly used method in those studies that included them. No study followed the accepted recommendation for using both HCA and FCA to cope with the uncertainty associated with these costs [84]. It is worth to mention one study that was excluded from the review because it only included the cost of lost productivity of two RDs (systemic sclerosis, sarcoidosis) apart from one non-rare disease (systemic lupus erythematosus) [85].

Limited perspectives, such as the patient perspective, can be informative but underestimate the actual burden of the disease for society [86]. However, they can be useful to highlight the economic burden of a disease for families as Eijgelshoven et al. did when estimated the cost of phenylketonuria (PKU) for patients and caregivers in the Netherlands [14]. They found that 99% of out-of-pocket costs were because of expenditure on low-protein food products, and that these costs were higher for severe PKU patients compared to mild PKU patients [14]; the actual economic burden of the disease for society as a whole is unknown. The perspective can have a dramatic influence on cost estimates depending on the health system, especially for those diseases which require caregivers dedication, those with high non-medical out-of-pocket costs or for countries where certain therapies or services are not reimbursed by the public health care system or a third payer [87, 88]. In those cases, the use of the perspective of society is especially critical. However, few studies report about the actual payers. For example, among the three most frequent diseases in our review (19 studies), only one study reported separately the cost paid by the patients and the cost paid by the government [48]. According to this study, the mean out-of-pocket costs per month related to ALS were $1871 (US dollars, year 2013), that is, 67.2% of mean household income in South Korea [48].

A top-down approach is possible when aggregated data are available [82]. We were unable to find studies with a top-down approach, except for the models, possibly because of the lack of aggregated data in the field of RDs. That is why authors probably tried to estimate the cost of RDs collecting data from primary sources such as patients or clinical charts. Although the use of large administrative databases makes it difficult to isolate medical costs directly attributable to the disease [86], hampering the estimation of incremental costs, and despite the risk of recall bias, the use of the bottom-up approach through retrospectively collecting data from patients and/or reviewing clinical charts is increasing, both in prevalent diseases [74, 82, 86, 89–91] and in RDs as we have seen in this review. Moreover, the use of real world data to estimate costs could be reinforced with new methods of analysis and the access to massive health data. One example of the potentiality of this is the study by Cai et al. [92], study that was not included in our review because of the date of publishing, where it was possible to estimate the cost of 23 RDs within a covered population of 61 million by means of the health information exchange system in Shanghai.

Ideally, COI studies should report both per-patient cost estimates and national COI estimates [5, 82, 89]. On the one hand, the annual per-patient estimation is useful to report costs in detail and to elucidate cost drivers [82]. However, they are not useful to know the actual dimension of the economic burden. On the other hand, reliable national estimates are difficult without high quality data on disease prevalence and on the distribution of features such as severity in the population with the disease. In our review we have observed that some authors reported the estimation of the national COI cautiously, probably because of a lack of quality data on prevalence of RDs [11, 34, 54, 72].

Cost estimates for the same disease can vary between studies for several reasons, apart from differences between countries in terms of unit costs, reimbursement policies or clinical practice. For example, the place of recruitment of the patients (primary care, specialised care, population-based samples) might be a source of heterogeneity [93], i.e. samples are more or less severely ill, and consequently tending to be more or less costly [74, 86, 89]. Zhou et al. [69] selected patients with haemophilia A aged 2–64 years from racially and geographically diverse regions in the USA, but they had to be recruited in six federally supported haemophilia treatment centres, had to receive at least 90% of their haemophilia care at this type of centre, and had to meet specific clinical characteristics. Therefore, this sample could not be fully representative of the whole population with haemophilia A. The authors estimated the burden for the 222 recruited patients which represent, according to the authors, 1% of individuals with haemophilia receiving care at these specialised centres in the USA. In their words, specialised centres facilitate recruitment but limit representativeness of the sample [69]. However, this fact might be irrelevant in some RDs given the level of specialised care that some RDs require.

There are several ways of reaching the patients, recruitment through patient’s organizations, by means of social media or accessing specific registries, databases or health centres [94]. Apparently, selecting patients from administrative databases could be more convenient than recruiting them from specialised centres in some circumstances [94]. But again, the drawback could be a potential selection bias. For example, those studies from the USA which use Medicare datasets, like many in our review, are estimating costs of patients aged 65 and above [82]. A community-based sample can facilitate the inclusion of a more representative group of patients, but the diagnosis of this cohort may depend on the reliability of the correct and complete codification of the diagnosis in the administrative data [94]. Moreover, theoretically the estimates should consider both the costs of undiagnosed patients [86] and diagnosed but untreated ones [89]. Otherwise there will be an underestimation of the actual economic burden [86]. If this occurs with common but poorly diagnosed chronic diseases such as depression or diabetes [86, 89], it is likely to be the case with RDs. In summary, the recruitment procedure and selection of the source of patients/data is critical to achieve a balance between representativeness and practicability.

Despite these issues, some authors can have a special interest in studying specific populations [65, 68] instead of mixed patients with the same disease, as is common to other diseases. Gidman et al. in their review identified papers which focused on specific subtypes of JIA or even patients treated with biological therapies [74]. This and other reviews found that biological therapies, new and expensive treatments, are associated with increased health care costs in more prevalent diseases like lupus, colorectal cancer and JIA [74, 82, 91]. Other cost drivers are ageing populations, as in atrial fibrillation [95], or productivity losses because of work capacity impairment, as in lupus [91]. All the above show the importance of studying the COI in RDs and non-rare diseases, their cost drivers, and evolution of the COI as well.

The methodology used to estimate the COI varies. In order to make comparison and interpretation possible, COI studies need to be more explicit about the scope, population characteristics, the costs included (perspective), how they were calculated, and so on [74, 80, 82, 84, 86, 89, 91, 93]. For example, cost categories should be presented in detail, and direct and indirect costs should be reported separately to facilitate identification of cost drivers for specific diseases [82, 84, 89].

This scoping review has three main limitations. Firstly, the search was not exhaustive. It would impossible with the current technology to search and review all the cost studies conducted in RDs as there are millions of RDs. Secondly, the definition of RDs is based on prevalence and a specific reference source was used to decide if one disease is rare or not [9], but we are aware of the fact that there are geographical differences in prevalence of many RDs. Thirdly, we did not assess the methodological quality of the studies. However, given that the aim was to show an illustrative overview of this topic, we believe our approach was sufficiently efficient. Finally, it was not our aim to overview the methods behind COI studies and how to conduct them, as there are many other articles on this topic [5, 81, 84, 90, 96], but to review a broad sample of COI studies in the field of RDs.

## Conclusions

In summary, our review found a paucity of COI analysis in RDs, in line with previous reviews [7, 97]. Most COI studies used prevalence-based estimations, cross-sectional design and bottom-up approaches, usually collecting data retrospectively and directly from patients or from clinical charts to estimate cost from the societal perspective. Apart from the common lack of data, COI studies of RDs are as feasible as COI studies of prevalent diseases. The main issue to address in the COI analysis of RDs is the lack of primary and/or aggregated data for most diseases that prevent a reliable estimation of the economic burden. Therefore, researchers have the mission of pursuing the generation of new knowledge where it does not exist, to comprehend the magnitude of the costs of RDs for society.

## Supplementary Information

**Additional file 1.** Main characteristics of the cost-of-illness studies included in the scoping review.

## Data Availability

Not applicable.

## Abbreviations

ALS: Amyotrophic lateral sclerosis
COI: Cost-of-illness
FCA: Friction cost approach
HCA: Human capital approach
JIA: Juvenile idiopathic arthritis
PKU: Phenylketonuria
RD: Rare diseases
